# The size-dependent morphology of Pd nanoclusters formed by gas condensation

**DOI:** 10.1039/c5nr06473b

**Published:** 2015-11-09

**Authors:** D. Pearmain, S. J. Park, A. Abdela, R. E. Palmer, Z. Y. Li

**Affiliations:** a Nanoscale Physics Research Laboratory , School of Physics and Astronomy , University of Birmingham , Edgbaston B15 2TT , UK . Email: Z.Li@bham.ac.uk

## Abstract

Size-selected Pd nanoclusters in the size range from 887 to 10 000 atoms were synthesized in a magnetron sputtering, inert gas condensation cluster beam source equipped with a time of flight mass filter. Their morphologies were investigated using scanning transmission electron microscopy (STEM) and shown to be strongly size-dependent. The larger clusters exhibited elongated structures, which we attribute to the aggregation, through multiple collisions, of smaller clusters during the gas phase condensation process. This was confirmed from the atomically resolved STEM images of the Pd nanoclusters, which showed smaller primary clusters with their own crystalline structures.

## Abstract 2

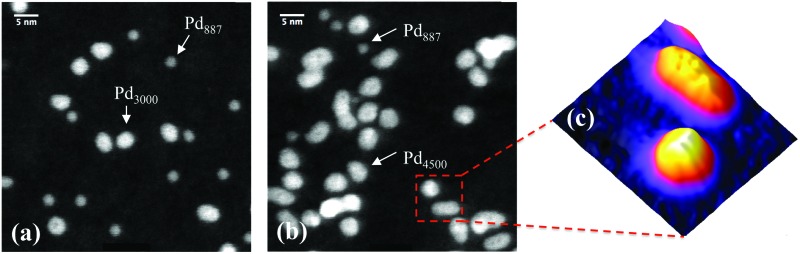
A scanning transmission electron microscopy (STEM) study of mass-selected Pd nanoclusters in the size range of 887 to 10 000 atoms, formed by inert gas aggregation, reveals a strong size-dependence of cluster morphology.

## 1 Introduction

In order to fully exploit the potential applications of cluster-based nanomaterials, it is necessary to gain full control of the cluster size, shape and structure.^1–7^ In terms of production, both wet chemical synthesis and physical methods have their advantages. In recent years, much progress has been made in gas phase approaches,^8^ notably gas condensation magnetron sputtering^9,10^ or laser vaporization.^11,12^ Gas phase synthesis allows for cluster mass selection prior to deposition on a support. Whilst the size (nuclearity) of gas-phase clusters can now be selected with atomic precision in some cases,^13^ the control of the shape and atomic structure of the deposited clusters remains a particularly challenging task. Experimental data in this field are scarce, partly due to the limited range of characterization techniques which can provide both size and morphology information.

We reported a systematic study which employs aberration-corrected scanning transmission electron microscopy (STEM) in high angle annular dark field (HAADF) mode to explore the size, shape and atomic structure of size-selected, positively-charged, Pd clusters in the size range of *N* = 887–10 000 atoms produced in a magnetron sputtering gas condensation cluster source.^9^ The clusters were size-selected by using a lateral time-of-flight mass spectrometer^14^ and then deposited on amorphous carbon coated Cu mesh TEM grids. We identified key parameters affecting the morphology of the clusters and established solid correlations between the size and morphology of the Pd clusters. Pd clusters occupy a special place in industrial catalysis.^7,15,16^ Our ultimate goal is to gain an insight into the mechanism of the formation of Pd clusters by gas phase condensation as a basis for the applications of such clusters in catalysis and beyond.

## 2 Results and discussion

**Fig. 1:**
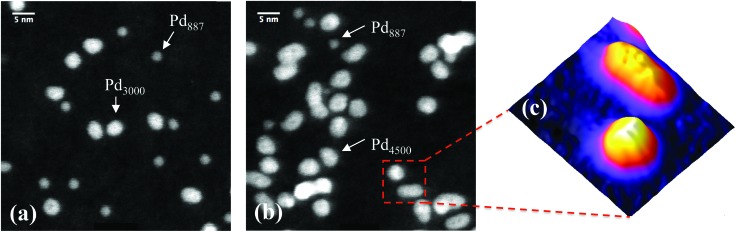
Typical HAADF-STEM images of (a) Pd_3000_ and Pd_887_ size-selected clusters, and (b) Pd_4500_ and Pd_887_. (c) shows a three-dimensional representation of the marked area (9 × 9 nm) shown in (b), which presents two Pd_4500_ clusters with elongated and circular projections.

Fig. 1 shows two STEM images of size-selected Pd_887_ clusters co-deposited with (a) Pd_3000_ and (b) Pd_4500_ clusters. These sizes are those selected by the mass filter, with ±2.5% mass resolution.^14^ The smaller clusters with circular projection in both Fig. 1(a) and (b) represent Pd_887_ clusters, while the larger Pd_3000_ and Pd_4500_ clusters are visibly distinguishable from their Pd_887_ counterparts in terms of their projected 2D sizes and their relatively higher intensities. Although the cluster loading has been kept constant, there are some local coverage variations. Most clusters are well separated. Examples of low and high coverage are shown in Fig. 1(a) and (b), respectively. Fig. 1(c) highlights two Pd_4500_ clusters, showing a three-dimensional representation of the HAADF intensity profile from the marked area in (b). Two Pd_4500_ clusters can be seen with different morphologies; one is elongated and the other is more circular.

**Fig. 2:**
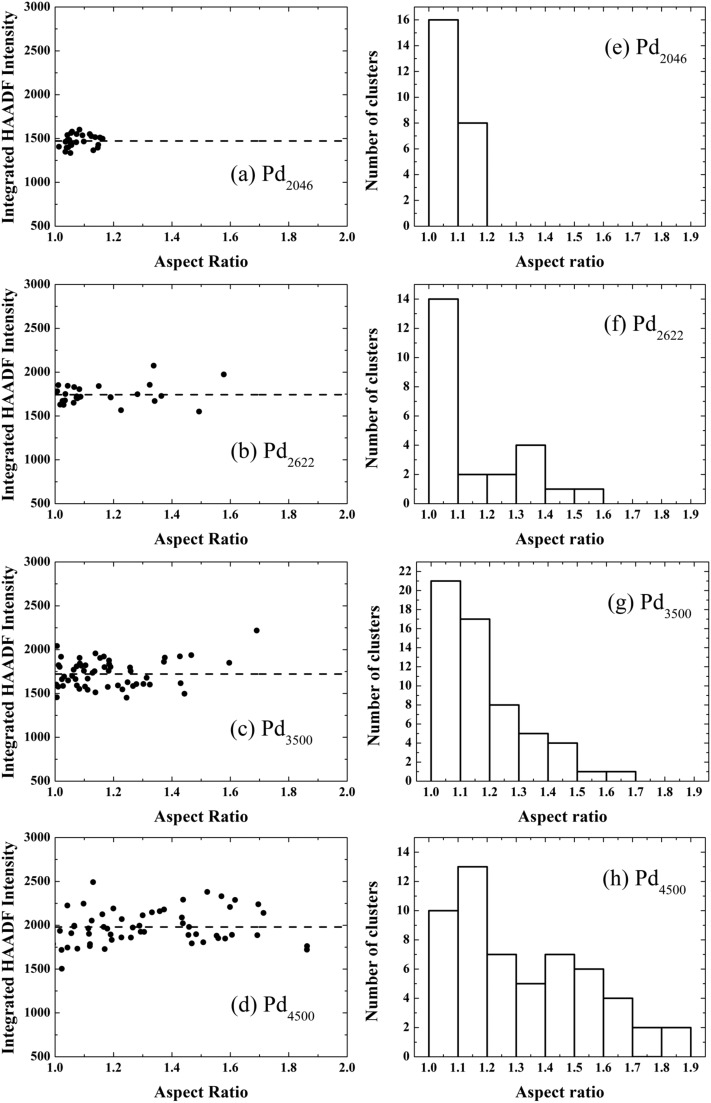
Integrated HAADF image intensity from individual (a) Pd_2046_, (b) Pd_2622_, (c) Pd_3500_, and (d) Pd_4500_ clusters as a function of their aspect ratio. Data points represent individual clusters of the respective size. The dashed lines show the average integrated HAADF intensity values for the corresponding cluster size. Histograms of cluster aspect ratios are presented in (e)–(h) for Pd_2046_, Pd_2622_, Pd_3500_, and Pd_4500_ clusters, respectively.

**Fig. 3:**
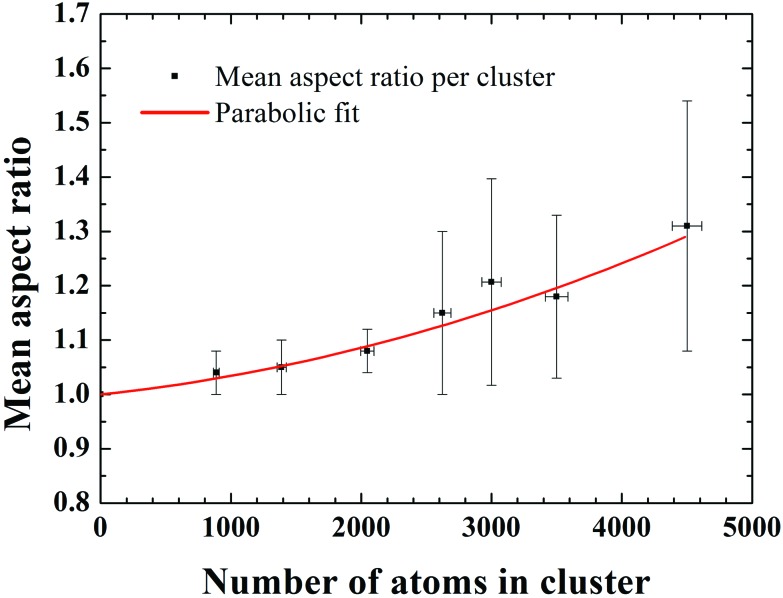
Mean aspect ratio as a function of cluster size in the range from Pd_887_ to Pd_4500_. The *x*-axis error bars are ±2.5% of the number of atoms in the clusters, determined by the mass resolution of the time-of-flight mass selector. The *y*-axis error bars represent standard deviations from the mean aspect ratios. The solid line is the result of the best parabolic-fit with *χ* ^2^ of 0.047, where the single atom, Pd_1_, with aspect ratio 1 was included.

To characterize quantitatively the size and shape of the clusters, we analysed the integrated STEM intensities from individual clusters of size 2046, 2622, 3500 and 4500 atoms. It has been shown previously that the integrated STEM intensity scales with the number of atoms within the clusters.^17–19^ Here, the Pd_887_ clusters were used as a mass standard for internal calibration.^17,19,20^ In Fig. 2(a)–(d) we plot the cluster intensity, after local carbon background subtraction, as a function of aspect ratio (measured as the ratio of the length of the long and short axis of the 2D projection of the cluster). Each data point represents an individual cluster measurement, while the dashed lines represent the average integrated intensity values associated with each cluster size. The independence of the mean intensity from the cluster aspect ratio confirms that the variation of 2D shape is not due to different cluster sizes. For example, if an elongated cluster was due to two individual clusters coalesced, the integral HAADF intensity would be roughly twice as that from the individual cluster (under the same microscopy conditions). The histograms of the aspect ratios for each cluster size plotted in Fig. 2(e)–(h) show that the distribution for the larger Pd clusters is much broader than the smaller ones. Fig. 3 shows a plot of the mean aspect ratio for each cluster size and shows an increasing deviation from circular projection (ratio 1) with the increasing cluster size. Evidently the large clusters are more likely to form elongated morphologies. The large ‘error bar’ for larger clusters is a sign of the size-dependent cluster morphology, indicating the clusters of varied shape and/or in varied orientation.

To understand this size-dependent behaviour, we first note that the data in Fig. 2(a)–(d) rule out the possibility that the elongated Pd clusters are formed by aggregation of two or more clusters on the support due to surface diffusion. If this were the case, one would find a change of the integrated STEM intensity with the aspect ratio of the clusters. In addition, the possibility of morphological change as a result of the impact on landing can also be ruled out, because the impact energy used in this study was low, ranging from 0.56 eV per atom for Pd_887_ to 0.05 eV per atom for Pd_10000_. Previous studies show clusters preserving their gas-phase shape in this so-called soft-landing regime.^21,22^ Thus we confidently attribute the size-dependent cluster morphology to the gas-phase formation process.

It is generally understood^23,24^ that metal cluster formation in an inert gas aggregation source involves several steps: first, liquid droplets are condensed from the vaporized (initially hot) target atoms in the cold buffer gas; these liquid clusters subsequently freeze into solid clusters. Further growth is either by atom addition or cluster–cluster collision. The cluster–cluster collision process is of particular interest for the formation of elongated clusters. If the kinetic energy of the colliding clusters is high enough, we may expect sintering to occur, probably leading to the formation of quasi-spherical large clusters. If not, then elongated Pd clusters can be envisaged as a result of the sticking together of the smaller cluster unit. The latter would happen more likely away from the target, where the gas temperature is reduced. The fact that we find a larger deviation of cluster shape as the size increases suggests that the energy barrier for reaching structural equilibrium states is higher for larger clusters. Our results and qualitative interpretation are in good agreement with the results of molecular dynamic (MD) simulations of the gas-phase condensation of Ni atoms (*N* ∼ 2000–8000),^23,24^ in which the simulated cluster shapes closely resemble our experimentally observed Pd clusters. The picture presented should apply to metal clusters of other elements too, although the size where elongated clusters set in will be material specific and also depend critically on the experimental conditions.

**Fig. 4:**
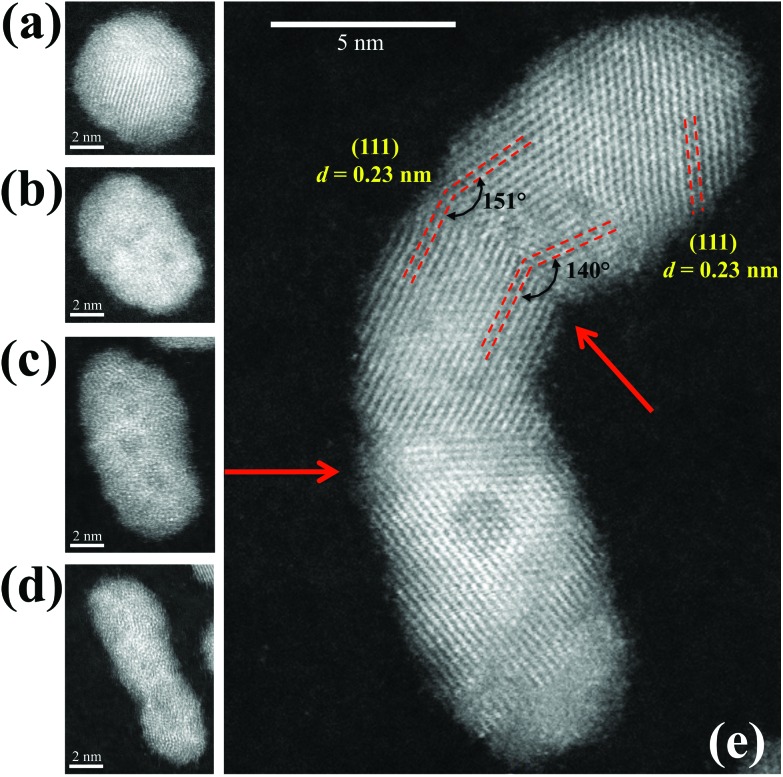
Aberration-corrected HADDF-STEM images of elongated Pd_10000_ nanoclusters. (a)–(d) Illustrate the range of aspect ratios for the cluster of the same size. (e) Reveals the local structure of one Pd_10000_ cluster. The arrows identify the junctions between the smaller constituent clusters. Lattice planes, and angles between them, and interplanar spacings are marked.

Further structural investigations into Pd_10000_ clusters were performed to gain atomic level insight into the elongated clusters. Fig. 4(a)–(d) show typical STEM images of Pd_10000_ clusters with varied elongation. Fig. 4(e) shows an atomically-resolved HAADF-STEM image of one of the Pd_10000_ clusters. It illustrates that the cluster formation is through aggregation of smaller component clusters and reveals the distinctive local structures of the constituent clusters. This Pd_10000_ cluster consists of three constituent clusters with two boundaries, as pointed out by the arrows. The shapes of the individual constituents can be seen clearly, together with the different crystalline orientations across the boundaries. At the boundary region between the upper two constituent clusters, the {111} planes (dashed lines) were observed in both clusters with the characteristic interplanar spacing of 0.23 nm of the Pd crystal,^25,26^ but with different orientations. It seems likely that the component clusters were already crystallized when aggregation took place and these individual crystalline structures retained. Detailed analysis revealed that the angle between two {111} planes at the upper boundary was in the range of 140° to 151°. This is likely caused by incomplete re-crystallization at the boundary. MD simulations of Pd nanoparticles showed that the interface region can melt during collision and then re-crystallize.^26^ In Fig. 4 we also see a region of low contrast circular shape in the lower part of the image, suggesting a “hollow” structure inside the cluster. We attribute this to a Kirkendall void.^27–30^

## 3 Conclusions

In summary, by a combination of mass-selected cluster deposition from an inert gas aggregation cluster source and advanced aberration corrected scanning transmission electron microscopy (STEM), a systematic study of Pd nanocluster structures has been carried out. A strongly size-dependent morphology of clusters with a size between 887 and 10 000 atoms has been unambiguously identified. The deviation from the spherical shape is much enhanced as the cluster size increases. Atomically resolved STEM images of an elongated Pd_10000_ nanocluster reveal that the cluster consists of a few smaller component clusters, which present individual crystalline structures. The re-crystallized twin structures were found to be a result from cluster–cluster collisions. The observations indicated that elongated Pd nanoclusters are mainly formed by the aggregation of component clusters due to cluster–cluster collisions in the gas phase. The work highlights the importance of the interplay between thermodynamic and kinetic factors in the morphology of clusters formed in the gas phase.

## 4 Experimental methods

Pd nanoclusters were synthesized using a magnetron sputtering, gas condensation cluster beam source.^9^ The gas pressure and sputtering power were fixed in this study. The positively charged Pd clusters were accelerated before size-selection with a lateral time-of-flight mass filter^14^ and deposition onto amorphous carbon coated Cu mesh TEM grids (Agar Scientific Ltd). The temperature in the condensation chamber was measured using a K-type thermocouple; this was electrically isolated, but exposed to the process gas. The temperature in the mid-position of the chamber was measured at ∼90 K.

Pd clusters were prepared in the size range of 887 to 10 000 atoms with a kinetic energy of 500 eV and each cluster size was co-deposited with Pd_887_, which was used as a mass standard.^17,19,20^ The mass filter resolution of *M*/Δ*M* ≈ 20, which is independent of mass, corresponds, for example, to a Pd_2046_ cluster containing 2046 ± 51 atoms. STEM images were obtained using an FEI Tecnai F20 electron microscope or a JEOL 2100F electron microscope with a spherical aberration corrector. Each microscope was operated with a field emission gun and an accelerating voltage of 200 kV. The incident probe size for the Tecnai F20 was ∼4 Å and for the JEOL 2100F it was around 0.8 Å. The high angle annular dark field (HAADF) detectors have inner and outer detection angles of 25 mrad to 127 mrad (Tecnai) and 61 mrad to 164 mrad (JEOL), respectively. The HAADF intensity over each cluster was analyzed using the software package ImageJ.^31^ Clusters overlapped with each other were excluded in the analysis. The cluster samples were stored in a vacuum, and only exposed to air briefly when transferring into the microscope. No post-treatment of the samples was performed in the present study.
